# Bilateral Empyema With Beta Hemolytic Group C Streptococcus and Streptococcus constellatus Co-infection Resulting From an Esophageal Perforation and Associated With Septic Shock, Diffuse ST Elevation, and New-Onset Atrial Fibrillation

**DOI:** 10.7759/cureus.57251

**Published:** 2024-03-30

**Authors:** Sabastain F Forsah, Derek Ugwendum, Nkeng Fuoching, Divine Besong Arrey Agbor, Kevin Villanueva, Nkafu Bechem Ndemazie, Gauvain Kankeu Tonpouwo, Nancelle Ndema, Keith Diaz, Jessie Saverimuttu, Jay Nfonoyim

**Affiliations:** 1 Internal Medicine, Richmond University Medical Center, Staten Island, USA; 2 Radiology, Richmond University Medical Center, Staten Island, USA; 3 Pulmonary and Critical Care, Richmond University Medical Center, Staten Island, USA; 4 Infectious Disease, Richmond University Medical Center, Staten Island, USA

**Keywords:** co-infection, diffuse st elevations, atrial fibrillation, group c streptococcus, streptococcus constellatus, septic shock, mediastinitis, esophageal perforation, bilateral empyema

## Abstract

Empyema is the collection of pus in the pleural cavity and most times, it occurs unilaterally. It is often associated with underlying pneumonia, but other causes have been identified as well. When it occurs after an esophageal perforation, which in itself is also rare, morbidity and mortality are even higher. Esophageal perforation can cause life-threatening complications due to its close proximity to the vital organs of the mediastinum, necessitating its timely diagnosis and aggressive management. Bacteria forming part of the normal esophageal and oral flora are the most common causative pathogens for empyema from an esophageal perforation. Streptococcus constellatus and group C Streptococci, though both rare and often not taken seriously, have been identified as individual causes of empyema. We present a case of a 58-year-old male who presented with a worsening cough, chest pain, and shortness of breath after choking on a fish bone. He was diagnosed with bilateral loculated empyema resulting from esophageal perforation with the pleural fluid culture isolating both group C streptococcus and Streptococcus constellatus. He also developed respiratory failure, mediastinitis, and septic shock. This case will enable physicians to take empyema caused by these bacteria seriously and also to include esophageal perforation as a differential diagnosis when a patient presents with bilateral empyema associated with chest pain and electrocardiographic changes.

## Introduction

Empyema is the collection of pus in the pleural cavity with 70% of cases developing due to an underlying pneumonia. It can also result from esophageal rupture, thoracic trauma, and post-thoracic surgeries. The United States registers about 32,000 cases of empyema per year and it is associated with high morbidity and mortality [1]. Bilateral empyema is rare with most cases of empyema being unilateral [2]. Among the bacteria implicated in community-acquired empyema, the most common are Streptococci. Bacteria constituting part of the normal flora of the oral cavity and the esophagus are more implicated in thoracic infections after an esophageal perforation, with anaerobes being the most commonly cultured [3,4]. In this case presentation, we discussed a patient who was diagnosed with culture-proven Streptococcus constellatus (S. constellatus) and Group C Streptococcus (GCS)-associated multiloculated bilateral empyema associated with septic shock and mediastinitis, resulting from an esophageal perforation. This case is unique because both esophageal perforation and bilateral empyema are rare and S. constellatus and GCS co-infection in an empyema have rarely been reported. Also, unlike in the past, empyema associated with normal esophageal bacteria flora should be taken seriously because they are increasingly being reported in empyema.

## Case presentation

A 58-year-old man with a past medical history of hypertension, type 2 diabetes mellitus, and more than 60-pack-year smoking history presented with a worsening cough, left-sided chest pain, and shortness of breath (SOB). Symptoms started with a non-productive cough for about one month. After he choked on a fish bone a week before presentation, he developed dysphagia which was associated with the worsening of his cough and new onset chest pain. He went to the emergency room (ER) when chest pain was worse during coughing episodes and associated with SOB, but he was discharged with supportive care after a chest x-ray showing a mild bilateral pleural effusion was the only significant finding. Two days later, he returned to the ER when his SOB and chest pain progressed with symptoms exacerbated when he laid down but improved when he sat up.

His initial vital signs showed a temperature of 98.4º F, heart rate of 155 beats per minute, respiratory rate of 32 breaths per minute, blood pressure of 205/112, and oxygen saturation of 84% on room air. He was put on noninvasive positive pressure ventilation (NIPPV) because he also had increased work of breathing.

On physical examination, the patient was alert, oriented, and diaphoretic. He was tachypneic, using accessory muscles of respiration with lung examination revealing decreased breath sounds in the bilateral lower lung fields. He was tachycardic and had irregularly regular heart sounds with no added sounds. The rest of the examination was unremarkable. Initial relevant laboratory investigations are shown in Table 1. Additionally, the respiratory viral panel was negative and blood gas on NIPPV showed a mild metabolic acidosis.

**Table 1:** Relevant initial laboratory results

Laboratory investigation (units)	Patient’s result	Reference Value
Hematology		
White cell count (K/UL)	15.8	4 – 11.2
Absolute neutrophil count (K/UL)	13.1	1.8 – 6.5
Absolute lymphocyte count (K/UL)	1.3	1.2 – 3.9
Hemoglobin (g/dL)	13.9	13.7 – 17.5
Biochemistry		
Sodium (mmol/L)	138	136 – 145
Potassium (mmol/L)	4.2	3.5 – 5.1
Blood urea nitrogen (mg/dL)	50	7 – 18
Creatinine (mg/dL)	2.6	0.7 – 1.3
Lactic acid (mmol/L)	3.8	0.5-2.2
High sensitive Troponins (n/L)	9.1	0 – 54

**Figure 1:**
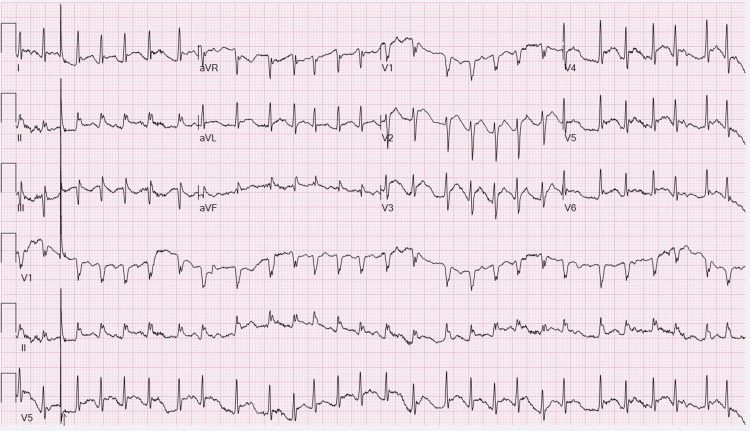
EKG showing atrial fibrillation with rapid ventricular response and diffuse S-T segment elevations.

An electrocardiogram (EKG) revealed a new onset of atrial fibrillation with rapid ventricular response and diffuse ST elevations (Figure 1).

The EKG findings in addition to the patient’s chest pain prompted an urgent cardiology consult for a suspicion of an acute myocardial infarction which was ruled out in favor of a pericarditis resulting from mediastinitis.

Initial chest x-ray was suggestive of bilateral pleural effusion and pulmonary edema. A diagnosis of hypertensive emergency with flash pulmonary edema was made for which blood pressure control was achieved with a nitroglycerin infusion. With suspicion of an underlying community acquired pneumonia, empirical antibiotics with azithromycin and ceftriaxone were started after collecting blood culture samples.

**Figure 2:**
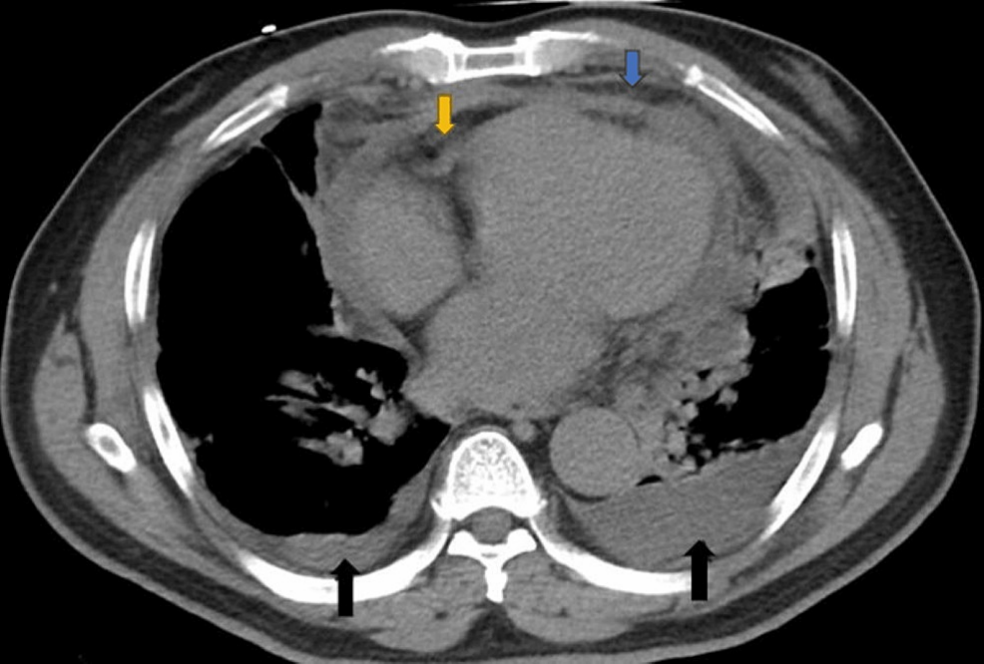
CT chest showing bilateral loculated pleural effusion, with left greater than right (black arrows), pericardial effusion (yellow arrow) and mediastinal fluid (blue arrows).

**Figure 3:**
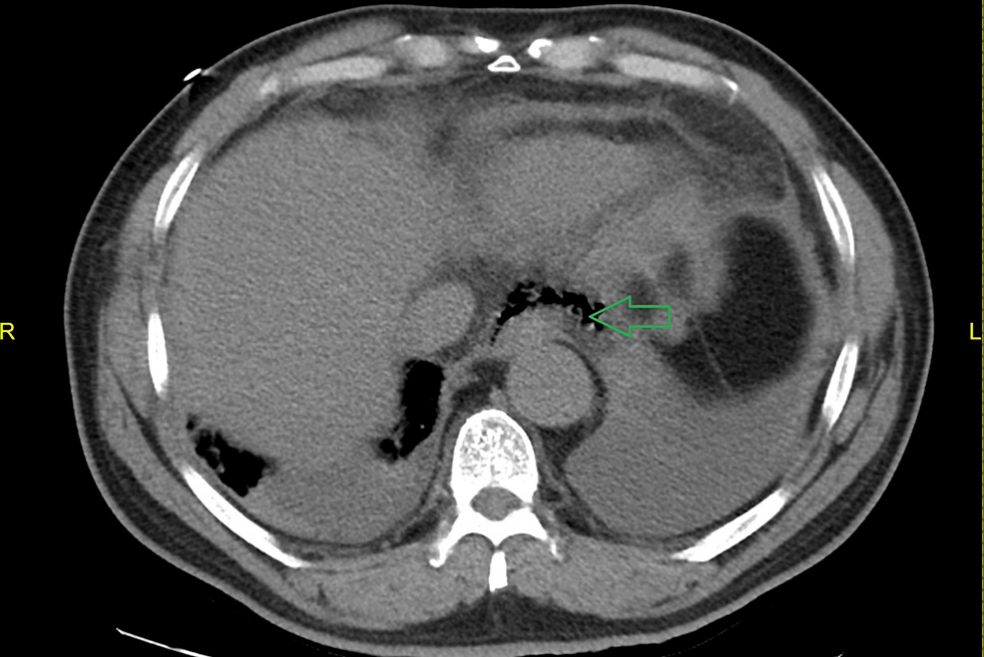
CT chest showing free gas surrounding the gastroesophageal junction in the lower aspect of the posterior mediastinum

Due to persistent respiratory distress even though patient was on NIPPV, a follow-up computed tomography (CT) of the chest was obtained which showed mild multiloculated right pleural effusion, moderate multiloculated left pleural effusion, mild pericardial effusion, moderate mediastinal free fluid (Figure 2). There was also a pneumomediastinum with free gas surrounding the gastroesophageal junction (Figure 3). There was no foreign body seen in the esophagus, no aortic dissection or pulmonary embolism.

CT findings prompted a cardiothoracic surgery consult. However, due to worsening respiratory distress, persistent tachypnea on NIPPV and a repeat blood gas revealing respiratory acidosis suggesting an impending respiratory failure, patient was intubated, mechanically ventilated, sedated and admitted in the intensive care unit.

Day 2 was marked by persistent high oxygen requirements, fever of 104ºF and hypotension requiring vasopressors. Antibiotics were changed to meropenem, vancomycin and clindamycin by the recommendations of the infectious disease specialist.

Management of bilateral empyema

A left chest tube was placed by general surgery, and it drained about 900mL of purulent pleural fluid within the first 12 hours. Pleural fluid analysis is shown in Table 2.

**Table 2:** Pleural fluid analysis

Pleural fluid parameters (units)	Patient’s result	Reference Value
White cell count (K/UL)	89	< 1
Lactate dehydrogenase (U/L)	>45000	-
Protein (g/dL)	2	< 1.5
Cholesterol (mg/dL)	48	<45
Triglyceride (mg/dL)	150	< 50
Amylase (U/L)	128	< 40

Further evaluation for other causes of increased pleural amylase was negative for pancreatitis, malignancy, tuberculosis and cirrhosis. Pleural fluid culture grew beta hemolytic GCS and S. constellatus. Blood cultures were negative while the culture of the endotracheal aspirate showed normal respiratory tract commensals. Clindamycin was stopped after seven days and ceftriaxone was added for streptococci in the pleural fluid, and vancomycin and meropenem were continued due to the persistence of the septic shock.

**Figure 4:**
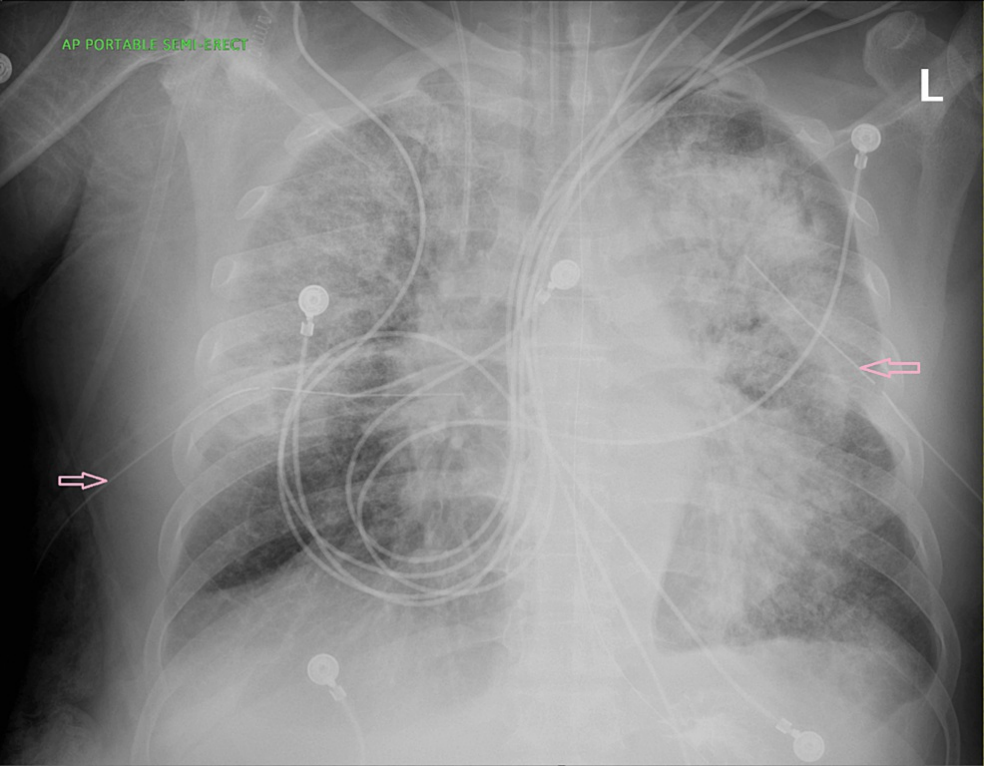
Chest x-ray showing bilateral chest tubes in place

The right sided empyema progressively got larger and a right chest tube was placed on day 8 of admission with an associated improvement in oxygen requirement. Cultures from the right empyema were negative probably because patient was already on antibiotics since admission (Figure 4).

The bilateral empyema progressively decreased in size as noted on daily chest x-rays done. The right and left chest tubes were removed seven and 15 days after their respective insertion. Ceftriaxone and vancomycin were discontinued after 20 days of treatment. 

Diagnosis and management of esophageal leakage

After the findings on CT chest, patient was placed on nothing by mouth (NPO), he was started on pantoprazole and was later placed on total parenteral nutrition. He was hemodynamically unstable for a CT chest with oral gastrografin to further investigate the esophageal perforation. Rather, methylene blue dye test was performed on day 3 to check for an esophageal leak. In that light, an orogastric tube (OGT) was placed in the upper esophagus, methylene blue dye was pushed through the OGT and the chest tube was monitor for extravasation. Numerous rounds were done and no extravasation was noted either immediately or later. CT esophagogram was only performed on day 14 which did not show any extravasation of gastrografin. The initial CT chest and pleural fluid findings were highly suggestive of esophageal perforation. There was no extravasation of contrast probably because either the perforation had healed spontaneously or it was a micro-perforation. Esophageal perforation was complicated by mediastinitis (atrial fibrillation, diffusely elevated ST segments on EKG and fluid in mediastinum), bilateral multiloculated empyema and septic shock.

Due to the patient’s failed daily liberation trials, he underwent a tracheostomy on day 25 of admission. Hospital course was also complicated by culture positive ventilator associated pneumonia and patient was treated for 14 more days with meropenem and Bactrim. Patient also had a bilateral pneumothorax from the chest tube, bifrontal cerebral infarction and severe anemia. On day 52 of admission, he was deemed stable for discharge to a short-term rehabilitation facility.

## Discussion

Esophageal perforation is very rare in the United States with about three out of 100,000 people diagnosed. Predisposing factors are the male gender and alcohol use [5]. The mortality even when therapy is initiated within 24 hours of esophageal perforation ranges from 10% to 25%, and it can rise to 60% when treatment is delayed for up to 48 hours [5,6]. Unfortunately, there is usually a treatment delay in more than 50% of cases because of the rarity at which esophageal perforation occurs and its nonspecific signs and symptoms [6]. The most common cause of esophageal rupture is iatrogenic, followed by spontaneous rupture, foreign body ingestion, trauma, and malignancy [3,6]. Spontaneous perforation of the esophagus, also referred to as Boerhaave’s syndrome can be induced by vomiting, straining, or coughing which causes an increase in intra-abdominal pressure. The increased intra-abdominal pressure is transmitted into the esophagus resulting in a tear [7].

The diagnosis of esophageal perforation can be achieved using a contrast esophagography which has the advantage in that, it can determine the location and size of the perforation. However, it has a false-negative rate of about 10%. The water-soluble gastrografin, though with lower sensitivity, is the preferred contrast in the context of esophageal perforation because it does not cause an inflammatory reaction in the mediastinum compared to barium contrast [8]. Computed tomography (CT) chest has a high sensitivity and specificity in diagnosing esophageal perforation and additionally, it can identify the complications arising from esophageal perforation like in our patient [9]. In an unstable patient like ours, the methylene blue swallow test can be performed at the bedside and an immediate discoloration of the chest tube content after swallowing methylene blue confirms the diagnosis of esophageal perforation. A delayed discoloration suggests a lymphatic leak into the pleural space. However, the limitation of this study is that the patient must have a drainable pleural effusion. Also, it cannot determine the location or size of esophageal leaks and adhesion formation may make it difficult to identify old esophageal leaks [10,11]. Our patient was diagnosed with esophageal perforation from the CT findings and pleural fluid analysis [11,12].

Surgery was the mainstay of treatment of esophageal rupture until recently when nonoperative management was gaining ground. Treatment includes intravenous fluid, nothing by mouth, total parenteral nutrition, broad-spectrum antibiotics, narcotic analgesics, non-operative management like in our patient, or surgical closure if indicated [13].

The complications of esophageal perforation are caused by bacteria and digestive enzymes gaining direct access into the mediastinum, causing inflammation and leading to the development of potentially life-threatening mediastinitis, empyema, and sepsis [6]. Our patient had mediastinitis characterized by mediastinal fluid, atrial fibrillation, and pericarditis [9,14].

The micro-organisms causing thoracic infections after an esophageal perforation are mostly those that constitute part of the normal flora of the oral cavity and the esophagus, and are therefore usually polymicrobial, with anaerobic bacteria most commonly isolated [4]. Among the bacteria implicated are the viridans streptococci with group C streptococci also reported [15,16]

S. anginosus (or S. milleri) is a group of viridans streptococci which include Streptococcus intermedius and S. constellatus [17]. The S. anginosus group is sometimes confused as belonging to other groups of Streptococci because they may react with C or G typing sera. However, they are different because they form small (<0.5 mm) colonies on sheep blood agar [17]. Unfortunately, empyema caused by S. constellatus has not been taken seriously firstly due to the special cultural conditions that make it difficult to be detected. Secondly, the fact that it is a commensal bacterium, is generally not considered a pathogen in respiratory infections [18]. However, S. constellatus has been associated with purulent infections in various organs of the body and it is being increasingly identified as a cause of empyema and should therefore be accorded attention in terms of diagnosis and management, especially in the context of esophageal perforation [15,17,19].

Group C Streptococci (GCS), with Streptococcus dysgalactiae subspecies equisimilis being predominant in humans, are gram-positive beta-hemolytic bacteria that also form part of the normal flora of the human upper airway, but infections have also been reported occasionally in individuals exposed to animals like horses [19]. These streptococci frequently cause infections of the throat, skin, and soft tissues in pregnant women [20] and have also been associated with lung pathology with complications including bilateral empyema like in our patient [16]. Our patient had bilateral empyema caused by a co-infection of S. constellatus and GCS which has been rarely reported.

Empyema caused by streptococcus species responds adequately to penicillin. However, anaerobic coverage is required regardless of negative cultures because anaerobes form part of the oral flora and are difficult to isolate in cultures [21]. An empyema requires drainage and tube thoracostomy is the most commonly used modality. Sometimes, fibrinolytics and mucolytics are used though it is currently not a standard of care. Surgery is the last resort and ranges from video-assisted thoracotomy to the more invasive open-thoracotomy as indicated [1,21].

Our patient had an esophageal rupture complicated by mediastinitis, bilateral empyema, and septic shock. He required respiratory and circulatory support and was treated with antibiotics, fluids, vasopressors, and parenteral nutrition while being kept NPO. The empyema was drained using a tube thoracostomy and though the patient’s clinical course was complicated by other medical conditions, he recovered and was discharged.

## Conclusions

Bilateral empyema is a major cause of morbidity and mortality with the risk even higher when it is associated with esophageal perforation and mediastinitis. The unique location of the esophagus makes esophageal perforation a life-threatening condition if not diagnosed and treated early. On the other hand, the rarity and nonspecific presentation of esophageal perforation delay diagnosis which increases the risk of mortality. Therefore, a detailed history, high index of suspicion, and aggressive management by a multidisciplinary team of intensivists, cardiothoracic surgeons, gastroenterologists, nutritionists, pulmonologists, and physical therapists are vital for the management of esophageal perforation and its complications. Physicians should take empyema caused by normal esophageal commensals seriously, especially in the context of an esophageal perforation. They should also think of esophageal perforation as a differential diagnosis when a patient presents with bilateral empyema associated with chest pain and electrocardiographic changes.
